# Prevalence and Antibiotic Resistance of *Bacillus* sp. Isolated from Raw Milk

**DOI:** 10.3390/microorganisms11041065

**Published:** 2023-04-19

**Authors:** Patryk Adamski, Zuzanna Byczkowska-Rostkowska, Joanna Gajewska, Arkadiusz Józef Zakrzewski, Lucyna Kłębukowska

**Affiliations:** Department of Industrial and Food Microbiology, Faculty of Food Science, University of Warmia and Mazury, Plac Cieszyński 1, 10-726 Olsztyn, Poland

**Keywords:** milk, *Bacillus* sp., antibiotic resistance

## Abstract

Milk, due to its diversity in terms of its nutritional content, is an important element of the human diet, as well as a good medium for the development of bacteria. The genus *Bacillus* contains ubiquitous aerobic, rod-shaped, endospore-producing gram-positive bacteria. Representatives of the *Bacillus cereus* group and the *Bacillus subtilis* group contribute to shortening the shelf life of milk and dairy products by degrading milk components and its additives. They also produce a number of heat-stable toxins and can cause a number of ailments, mainly in the digestive system. The aim of this research was to identify *Bacillus* sp. strains isolated from raw milk and to determine their antibiotic resistance. Strains isolated from raw milk samples (*n* = 45) were identified by MALDI-TOF MS. Ninety strains of *Bacillus* sp. were identified, for which the antibiotic resistance phenotype was determined. A total of 90 strains of *Bacillus* were classified in five groups (the *Bacillus cereus* group (*n* = 35), *B. licheniformis* (*n* = 7), the *B. subtilis* group (*n* = 29), *B. pumilus* (*n* = 16), and *Bacillus* sp. (*n* = 3). All isolates were susceptible to chloramphenicol and meropenem. The antibiotic resistance profiles of the tested groups of *Bacillus* spp. differed from each other, which is of particular concern in relation to multidrug-resistant representatives of the *B. cereus* group resistant to cefotaxime (94.29%), ampicillin (88.57%), rifampicin (80%), and norfloxacin (65.71%). Our study provides data on the prevalence and antibiotic sensitivity of *Bacillus* sp. In raw milk, suggesting a potential risk to health and the dairy industry.

## 1. Introduction

Milk, due to its diversity in terms of its nutritional content, is an important element of the human diet and at the same time a good medium for the development of bacteria [1]. Milk that has not been processed is an important source of bacterial infection. Non-compliance with the hygiene standards of its acquisition makes it difficult to avoid the contamination of milk with microorganisms [2]. The level of microbiological contamination of milk depends on several factors such as animal health, farm sanitary conditions, milking hygiene, and milk storage temperature [3,4]. In most cases, unless the animal is suffering from a mammary gland infection or systemic disease, the milk produced by a mammary gland should not contain bacteria, although it is easily contaminated with microbes living on the animal’s skin during milking. In Europe, according to the Commission Regulation (EC) No. 1662/2006 of 6 November 2006, raw milk should not contain >1 × 10^5^ microorganisms per mL [5]. Good quality raw milk determines its technological suitability and the appropriate quality and shelf life of dairy products. The presence of pathogens in milk is a potential threat to public health, especially among consumers of raw milk [6,7].

The dominant microbiota of chilled raw milk is psychrotrophic bacteria, capable of producing undesirable proteolytic and lipolytic enzymes, causing adverse changes in dairy products [3,4,8]. Among the most significant bacteria causing spoilage of dairy products are bacteria belonging to the genus of psychrotrophic *Bacillus*.

The genus *Bacillus* is a ubiquitous aerobic, rod-shaped, endospore-producing gram-positive bacteria. The most important *Bacillus* species contaminating raw milk are *Bacillus cereus*, *Bacillus licheniformis*, and *Bacillus pumilus* [9]. *B. cereus* and the listed members of the *B. subtilis* group contribute to shortening the shelf life of milk and dairy products by degrading milk components and produce a number of thermostable toxins that can also cause digestive system ailments [10,11,12]. According to literature data, a *B. cereus* count above 5.0 CFU/mL may cause taste and flavour defects in pasteurized milk. With a higher presence of *B. cereus*, the products show defects, such as sweet curd and bitty cream, due to high proteolytic activity and lecithinase production [13]. Some *B. cereus* strains can potentially grow at 8 °C and below to a concentration that may be detrimental to human health [14]. However, most strains of *Bacillus* spp. are not pathogenic for humans, but some may infect humans incidentally. *B. cereus* enterotoxins were associated with the highest number of foodborne outbreaks among bacterial toxins, exceeding outbreaks caused by *Clostridium perfringens* and *Staphylococcus aureus* [15]. It is most commonly associated with gastrointestinal disease manifested as vomiting. Rarely, it can also cause eye infection, meningitis, pneumonia, periodontitis, necrotizing fasciitis, and osteomyelitis [9,16,17,18,19].

In addition to the defects of dairy products causing economic losses in the food industry, contaminated milk can cause infections and contribute to the widespread problem of antibiotic resistance due to the extensive use of pharmaceutics in cattle farming. Bacterial resistance to antimicrobial substances may be intrinsic, acquired, and/or adaptive [20]. Selective pressure exerted on microorganisms using antimicrobial agents has so far been defined as the main mechanism of antibiotic resistance. Antibiotics affect susceptible bacteria, while resistant ones survive by passing on the resistance gene to daughter cells [21]. Another form of transmission of resistance genes to microorganisms is horizontal gene transfer (HGT) [22].

Antimicrobial agents at low doses (sublethal or subtherapeutic) in the form of residues in feed might also be factors influencing bacterial antibiotic resistance, as they are able to induce genetic and phenotypic variability in exposed bacteria [23,24]. Thus, antibiotic residues in the food chain can cause antibiotic resistance to transfer not only in pathogens but also in commensal bacteria or lactic acid bacteria [25,26,27].

Although *Bacillus* spp. are often isolated from milk and dairy products [28], and although this industry, based on animal production, has a prominent place in the process of the development and dissemination of drug resistance in the environment, there are few reports on the antibiotic resistance profiles of *Bacillus* spp. isolated from milk. Therefore, the aim of this research was to identify *Bacillus* spp. strains isolated from raw milk and to determine their antibiotic resistance.

## 2. Materials and Methods

### 2.1. Isolation of Bacillus spp. Strains

Raw milk samples (*n* = 45) obtained from farms located in the Warmia-Masuria Province were subjected to analysis. All milk specimens were transported to the laboratory immediately after collection. Ten milliliters of each raw milk sample was pasteurized for 15 min at 80 °C to remove non-sporing bacteria. Next, the sample was streaked in Mannitol Egg Yolk Polymyxin Agar (MYP) (Merck, Darmstadt, Germany) and nutrient agar (Merck, Darmstadt, Germany), then incubated for 48 h at 30 °C.

### 2.2. Identification by MALDI-TOF

Strains were identified using VITEK^®^MS (bioMérieux, Marcy l’Etoile, France) according to the manufacturer’s protocol, described previously [29]. Briefly, characteristic colonies from the MYP agar and nutrient agar plates were cultured for 48 h at 30 °C on Tryptic Soy Agar (TSA) (Merck, Darmstadt, Germany). After incubation, a small portion of bacterial colonies were transferred to the MALDI target plates. Then, 1 μL of MALDI matrix VitekMS-CHCA (α-Cyano-4-hydroxycinnamic acid) (bioMérieux, Marcy l’Etoile, France) was added to the spots and then dried at room temperature. Strains were analyzed by the VITEK^®^MS v2.0 MALDI-TOF mass spectrometry systemV2.0 (RUO; SARAMIS version 4.13) databases (bioMérieux, Marcy l’Etoile, France). We considered the effectiveness of the MALDI-TOF identification method when the significance level was ≥90% [30]. For calibration and quality control, *Escherichia coli* ATCC 8739 was used.

### 2.3. Phenotypic Antibiotic Resistance Analysis

Antimicrobial susceptibility was determined using the Kirby–Bauer disc diffusion method according to the standard procedure described by EUCAST (European Committee on Antimicrobial Susceptibility Testing) [31]. Twelve antibiotics (Oxoid, UK) commonly used in human and animal infections were used. The selected antibiotics belong to eleven classes of antimicrobials: aminoglycosides: gentamicin (CN, 10 µg) and amikacin (AK, 30 µg); aminopenicillins: ampicillin (AMP, 10 µg); carbapenems: meropenem (MEM, 10 µg); lincosamides: clindamycin (DA, 2 µg); macrolides: erythromycin (E, 15 µg); glycopeptides: vancomycin (VA, 30 µg); third-generation cephalosporins: cefotaxime (CTX, 30 µg); phenicols: chloramphenicol (C, 30 µg); rifampicins: rifampicin (RD, 5 µg); sulfonamides–trimethoprims: trimethoprim/sulfamethoxazole (SXT, 25 µg); and fluoroquinolones: norfloxacin (NOR, 10 µg).

Firstly, suspensions in sterile saline (0.9%) were prepared from bacterial colonies on TSA (Merck, Darmstadt, Germany) cultured for 24 h until they reached a 0.5 McFarland standard concentration. A sterile swab was used to inoculate the suspension on Mueller–Hinton agar (Merck, Darmstadt, Germany) [32]. Antibiotic discs were then placed on the plates and incubated at 37 °C for 24 h. The inhibition zone diameters were recorded after the incubation period. Strains were categorized as resistant (R), intermediate-resistance (I), and susceptible (S) according to the criteria in EUCAST [31] for *Bacillus* sp. Additionally, due to the lack of standards for antibiotics not included in EUCAST for *Bacillus* sp., the standards for staphylococci were used [32]. *Staphylococcus aureus* 29213 was used as quality control (QC) for most of the tested antibiotics, *Enterococcus feacalis* ATCC 29212 was used as QC for vancomycin, and *Escherichia coli* ATCC 29212 as QC for meropenem [31].

The multiple antibiotic resistance (MAR) index was calculated for each isolate as: number of antibiotics to which the isolate is resistant/total number of antibiotics against which the isolate was tested [18,33]. In this study, we defined multidrug resistance (MDR) as resistance to at least one antibiotic from three or more classes of antibiotics [33].

### 2.4. Statistical Analysis

Statistical analyses were performed using GraphPad Prism software version 8.0 (GRAPH PAD Software Inc, San Diego, CA, USA) and *p* ≤ 0.05 was considered significant.

## 3. Results

### 3.1. Isolation and Identification of Bacillus spp.

**Figure 1:**
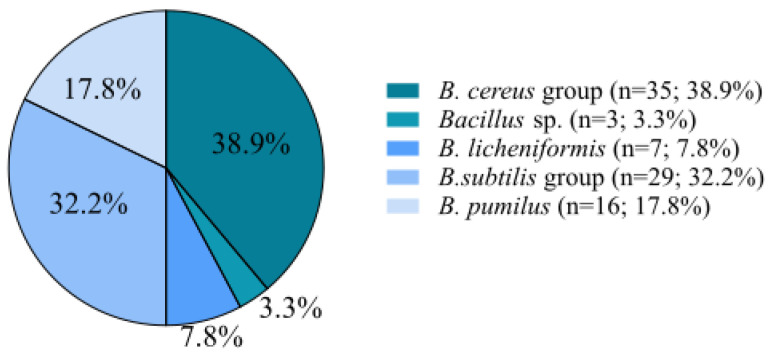
Distribution of *Bacillus* spp. isolates in different farm types (*n* = 90).

From 45 raw milk samples, a total of 90 strains of *Bacillus* sp. were isolated. Their identification using MALDI-TOF allowed for them to be classified into five groups: the *Bacillus cereus* group (38.9%; *n* = 35), *B*. *licheniformis* (7.8%; *n* = 7), the *B*. *subtilis* group (32.2%; *n* = 29), *B*. *pumilus* (17.8%; *n* = 16), and *Bacillus* sp. (3.3%; *n* = 3). We found that the *B. cereus* group was dominant (Figure 1).

### 3.2. Antibiotic Resistance Pattern

**Figure 2:**
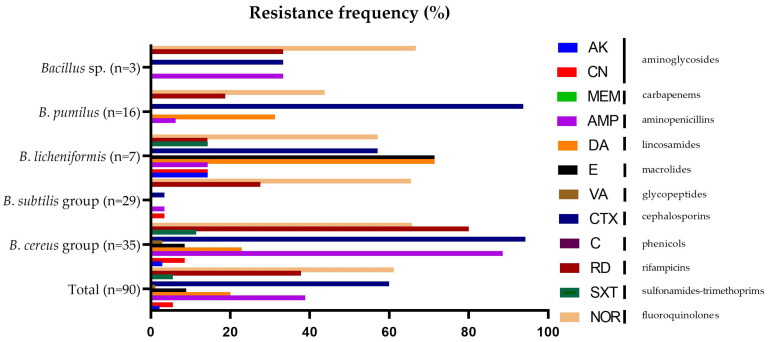
Antimicrobial resistance frequency (%) for the studied *Bacillus* strains (*n* = 90) isolated from raw milk. The isolated strains belonged to the following groups: *Bacillus* sp., *B*. *subtilis* group, *B*. *cereus* group, or/and represented species *B*. *licheniformis* and *B*. *pumilus*. Abbreviations: CN—gentamicin, AK—amikacin, AMP—ampicillin, MEM—meropenem, DA—clindamycin, E—erythromycin, VA—vancomycin, CTX—cefotaxime, C—chloramphenicol, RD—rifampicin, SXT—trimethoprim/sulfamethoxazole, and NOR—norfloxacin.

The results of our study showed that the tested *Bacillus* spp. showed resistance to antimicrobial agents from different classes (Figure 2). The resistance frequency of the tested antibiotics among all the investigated strains ranged from 0 to 94.29%. All isolates were susceptible to chloramphenicol and meropenem, and most of the studied strains were susceptible to vancomycin (98.89%), amikacin (97.8%), gentamicin (94.44%), trimethoprim/sulfamethoxazole (94.44%), erythromycin (91.12%), ampicillin (61.11%), and rifampicin (54.44%). The highest resistance was observed for norfloxacin (61.11%) and cefotaxime (60.00%) (Figure 2). Resistance profiles differed between the groups.

All of the *B*. *subtilis* group strains (*n* = 29) were susceptible to chloramphenicol, meropenem, amikacin, vancomycin, trimethoprim/sulfamethoxazole, clindamycin, and erythromycin, and most of the studied strains were susceptible to cefotaxime (96.55%), gentamicin (96.55%), ampicillin (96.55%), and rifampicin (72.41%). Within the *B*. *cereus* group, all of the studied strains were susceptible to chloramphenicol and meropenem, and most of them were susceptible to amikacin (97.14%), vancomycin (97.14%), gentamicin (91.43%), and sulfamethoxazole (88.57%). All of the *B*. *licheniformis* strains (*n* = 7) were susceptible to chloramphenicol, meropenem, and vancomycin, and most of the studied strains were susceptible to amikacin (85.71%), gentamicin (85.71%), trimethoprim/sulfamethoxazole (85.71%), rifampicin (85.71%), and ampicillin (85.71%). *B*. *pumilus* strains (*n* = 16) were completely susceptible to chloramphenicol, meropenem, amikacin, trimethoprim/sulfamethoxazole, vancomycin, erythromycin, and gentamicin, and most of the studied strains were susceptible to ampicillin (93.75%), rifampicin (81.25%), clindamycin (68.75%), and norfloxacin (56.25%). Non-identified *Bacillus* strains (*n* = 3) were susceptible to chloramphenicol, meropenem, amikacin, erythromycin, trimethoprim/sulfamethoxazole, vancomycin, clindamycin, and gentamicin, and most of the studied strains were susceptible to ampicillin (66.67%), cefotaxime (66.67%), and rifampicin (66.67%). Based on the Kruskal–Wallis ANOVA, it was noted that the resistance profile is species dependent for five antibiotics: ampicillin (*p* < 0.000001), clindamycin (*p* = 0.00035), erythromycin (*p* = 0.034479), cefotaxime (*p* < 0.000001), and rifampicin (*p* = 0.000014).

Most of the *B*. *cereus* group strains were resistant to cefotaxime (94.29%), ampicillin (88.57%), rifampicin (80%), and norfloxacin (65.71%). The *B*. *subtilis* group strains were resistant to norfloxacin (65.52%). Most of the studied *B. licheniformis* strains (*n* = 7) were resistant to erythromycin (71.43%), clindamycin (71.43%), cefotaxime (57.14%), and norfloxacin (57.14%). Meanwhile, most of the studied *B*. *pumilus* strains (*n* = 16) were found to be resistant to cefotaxime (93.75%). Two of the three tested non-identified *Bacillus* sp. strains (*n* = 3) were resistant to norfloxacin (66.67%).

**Figure 3:**
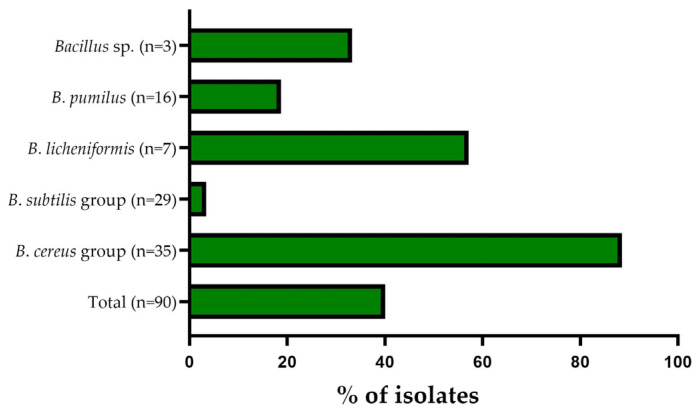
Multidrug-resistance frequency (%) for the studied *Bacillus* strains (*n* = 90) isolated from raw milk. The isolated strains belonged to the following groups: *Bacillus* sp., *B. subtilis* group, *B. cereus* group, or/and represented species *B. licheniformis* and *B. pumilus*.

Among all the *B. cereus* group isolates, 31/35 (88.57%) were found to be multidrug resistant (Figure 3). Within the *B. cereus* group, the multiple antibiotic resistance (MAR) index was found to range from 0.08 to 0.66 and the overall mean was 0.34. Among the *B. subtilis* group, one of the 29 strains (3.45%) was defined as multidrug resistant (Figure 3), with the multiple antibiotic resistance (MAR) index calculated as 0.25. However, 57.14% (4/7) strains of *B*. *licheniformis* were defined as multidrug resistant (Figure 3). The multiple antibiotic resistance (MAR) index among isolates was found to range from 0.17 to 0.50, with an overall mean of 0.33. Within *B*. *pumilus* strains, 18.75% (3/16) were specified as multidrug resistant (Figure 2). The multiple antibiotic resistance (MAR) index of the studied strains was found to range from 0.08 to 0.33 and the overall mean was 0.13 (Figure 3). Among non-identified *Bacillus* strains, 33.33% (1/3) was defined as multidrug resistant (Figure 3), with the multiple antibiotic resistance (MAR) index calculated as 0.33.

Our results showed 26 antibiotic resistance profiles for all isolates. Among the tested strains, 12.2% (11/90) belonging to *B. licheniformis* (*n* = 1), *B. pumilus* (*n* = 1), the *B. subtilis* group (*n* = 8), and *Bacillus* sp. (*n* = 1) were sensitive to all tested antibiotics. Next, 22.2% (20/90) were resistant to only one antibiotic, mainly norfloxacin (14/90; 15.6%). Moreover, 20% (18/90) of the strains were resistant to two antibiotics, mainly rifampicin and norfloxacin (7/90; 7.8%). The other strains showed resistance to three or more antibiotics. The most resistant strain was resistant to eight antibiotics (AK, AMP, DA, E, VA, CTX, RD, NOR). The results showed that two antibiotic resistance profiles occurred most frequently: AMP–CTX–RD (11/90; 12.2%) and AMP–CTX–RD–NOR (11/90; 12.2%) (Table 1).

## 4. Discussion

The presence of *Bacillus* sp. in milk can cause spoilage in dairy products and food poisoning due to the enterotoxins produced by these microorganisms. *B. cereus* and other *Bacillus* spp. are common etiologic agents of foodborne diseases worldwide. Global statistics on food poisoning caused by *B. cereus* are underestimated due to the occurrence of vomiting symptoms similar to those of *S. aureus* poisoning and diarrheal symptoms similar to those caused by *Clostridium perfringens* type A. A significant proportion of people affected by food poisoning caused by *Bacillus* sp. do not seek medical help due to the short duration of the symptoms [34]. Species other than *B. cereus* are not indicated in clinical diagnostics as the etiological factors of food poisoning. However, studies have confirmed the production and action of thermolabile toxins and cereulide-like toxins produced by *B. circulans*, *B. lentus*, *B. subtilis*, *B. licheniformis*, *B. pumilus*, and *B. amyloliquefaciens*. Significantly, outbreaks caused by *B. pumilus* and *B. subtilis* are often wrongly assigned to *B. cereus* [35]. *Bacillus* spp. are also known to form biofilms resistant to commonly used technological processes, which is a serious problem for the dairy industry. *Bacillus* spp. are generally capable of producing extracellular or intracellular thermostable proteo- and lipolytic enzymes that are involved in the spoilage of milk and dairy products, leading to unfavorable organoleptic changes [34,36,37].

Some non-toxic strains of *Bacillus* sp. are used as probiotics in animal feed and additives in the food industry, including the dairy industry. In recent years, *Bacillus* spp. have gained interest in research on functional foods related to human health due to their increased tolerance and ability to survive in the unfavorable environment of the digestive tract. In addition, the bacteria are more stable during the processing and storage of foodstuffs and even pharmaceutical preparations. However, due to the their potential pathogenicity, the safety of individual strains of the genus *Bacillus* should be studied and a deeper analysis should be carried out in order to select the strains used as probiotics [38].

Hornik et al., based on the conducted research, found that *Bacillus* sp. ranges from 10 to 17% of the milk microbiome. The results differ depending on the origin of the sample collection [39]. Studies show that *B. licheniformis*, along with *B. cereus*, is one of the most widespread *Bacillus* species found in raw milk and in the entire milk-processing chain [35]. In addition, previous studies [40] indicate that it is the dominant species of spore-forming bacteria (68%) found in powdered skimmed milk. Other authors indicate that *B. licheniformis* was the second most common species of spore-forming bacteria detected in a study of 28 milk powder samples from 18 different countries, with a total prevalence of 39.2% [41]. In our research, *B. licheniformis* accounted for only 7.78% (7/90) of *Bacillus* sp. strains isolated from raw milk. Referring to the work of Heyndrickx and Scheldeman [42], *B. licheniformis* was the dominant species, found in greater abundance over *B. subtilis* and *B. pumilus* in pasteurized milk and its products, which is inconsistent with our results. *B. licheniformis* strains accounted for 7.78% (7/90), while *B. pumilus* and the *B. subtilis* group accounted for 17.77% (16/90) and 32.22% (29/90), respectively. Nieminen et al. [37] identified 21.74% (5/23) strains of *B. pumilus* in a study of milk from cows with mastitis.

In addition, in a study conducted by Sarkar and Kumari [43], strains of the *B. cereus* group were isolated from six out of eight different dairy products sold in India. Their occurrence in cheese, ice cream, powdered milk, and pasteurized/sterilized milk was relatively high (33–55%). In the work of Rahnam et al., *B. cereus* was present in 60% of raw milk samples, constituting 75.00% (34/44) of all *Bacillus* sp. isolates [28]. The results obtained in this study show that species from the *B. cereus* group are the most common representatives of *Bacillus* sp. in raw milk, constituting 38.89% (35/90) of isolated *Bacillus* sp. strains.

The World Health Organization warns that the increasing prevalence of antibiotic resistance is a serious threat and one of the greatest public health problems of the 21st century [44]. Bacteria that have developed mechanisms of resistance against individual antibiotics, using the horizontal gene transfer (HGT), can transfer their resistance genes to other bacteria, including the microbiome of the human digestive tract [39], intensifying the problem of antibiotic resistance. Toth et al. [26] confirm that ARGs can be found in raw milk. In addition, the use of antimicrobials is widespread in the farm environment, which contributes to the phenomenon of the milk microbiome acquiring resistance to these substances. Raw milk that has not undergone heat treatment is a convenient environment for microbial proliferation, and this affects the amplification of ARGs. Their intensity increases the risk of horizontal gene transfer.

Gundogan and Avci [45] reported that *B. cereus* isolates recovered from raw milk and dairy products in Turkey samples were resistant to ampicillin (91.1%) and trimethoprim/sulfamethoxazole (27.8%). Chang et al., studying resistance in raw and pasteurized milk, indicated resistance to ampicillin (96.00%) and trimethoprim/sulfamethoxazole (10.40%) strains of *B. cereus* [46]. Our results show a similar level of resistance to ampicillin (88.57%) and trimethoprim/sulfamethoxazole (11.43%). Hu et al. [47] isolated *B. cereus*, *B. subtilis*, *B. licheniformis*, and *B. pumilus* from food samples from local markets and restaurants. All the tested strains of *B. cereus* showed resistance to ampicillin. In contrast, none of the isolates showed resistance to rifampicin and vancomycin. However, our research showed that among the tested strains, only one strain from the *Bacillus cereus* group was resistant to vancomycin, and 80% were resistant to rifampicin. Kong et al. [48] identified *B*. *cereus* in 26.37% (159/603) samples of meat and meat products. All of the studied strains showed resistance to ampicillin and most of them were resistant to rifampicin (86.29%). In this study, 88.57% (31/36) strains of the *B*. *cereus* group were resistant to ampicillin and 80.00% (28/35) showed resistance to rifampicin. In contrast, most strains were susceptible to gentamicin, chloramphenicol, and trimethoprim-sulfamethoxazole, which is supported by our study. In their study, all of the studied strains were resistant to at least three classes of antibiotics, with the multiple antibiotic resistance (MAR) index ranging from 0.15 to 0.50.

In another study, Yang et al. [49] described the antibiotic resistance profile of common bacteria strains isolated from various environments (water, digestive tract, soil, animal products). They indicated that a *B*. *subtilis* isolate (*n* = 1) was resistant to ampicillin and gentamicin. However, the results obtained in this study show high sensitivity to ampicillin (96.55%) and gentamicin (96.55%) among the tested strains of the *B. subtilis* group (*n* = 29). It is worth noting that *B. subtilis*, despite the confirmed cases of contamination of dairy products and posing a health risk to consumers [35,50], still remains marginalized in terms of its presence in dairy products.

Pasteurization is carried out to kill unwanted microorganisms present in raw milk. There is a risk that this process will not eliminate the spores produced by bacteria, including those of the *Bacillus* genus. Zhui et al. [51] isolated strains of *Bacillus* sp. from pasteurized milk. In their study, 80% of the strains showed resistance to ampicillin; in our study, 35/90 strains (38.88%) were resistant to ampicillin. In the cited study, 10/114 (8.77%) strains were resistant to trimethoprim/sulfamethoxazole, while our study showed 5/90 (5.55%) strains resistant to this agent. The authors indicate 8/114 (7.01%) strains resistant to clindamycin and 2/114 resistant to erythromycin. In our case, the values of resistance to these two substances were 20.00% (18/90) and 5.55% (5/90), respectively.

The *B. subtilis* group also includes the *B. licheniformis* and *B. pumilus* species, however, due to their frequency of occurrence, they were included in the study separately. Jeong et al. showed more than four times the breakpoint resistance to clindamycin in 70.2% of 74 strains of *B. licheniformis* derived from fermented soybean products [52]. Hu et al. indicated 100% susceptibility to gentamicin of the tested strains of *B. pumilus* isolated from dairy products, probiotics, fermented food, rice products, raw or cooked meat, fermented soy beverage, and snacks from different local markets and restaurants in China [47]. In this study, all of the studied *B*. *pumilus* strains (*n* = 16) were susceptible to gentamicin and the majority of them were susceptible to clindamycin (68.75%).

The study showed that a high percentage (40/90 (40.11%)) of the tested isolates were multidrug resistant (resistant to at minimum three antibiotics from different chemical classes of antibiotic), with 100% of isolates having a MAR index >0.20. MAR index values higher than 0.2 suggest a high level of antibiotic resistance among strains isolated from milk [18,33]. Nevertheless, it is worthy of attention that in our study, 77.50% (31/40) of the multidrug-resistant *Bacillus* strains were from the *B. cereus* group. The high incidence of multidrug-resistant strains indicates the need to introduce an antibiotic surveillance program in the dairy industry. In addition, determination of the MAR index may be useful, especially in cases of nosocomial infections, allowing for the introduction of effective antibiotic therapy [18].

Previous studies focused mainly on determining the presence, identifying sources of contamination, and characterizing *B. cereus* as one of the most important microorganisms affecting the quality and safety of dairy products [12,16,46]. Nevertheless, the presented studies also raise the aspect of antibiotic resistance of other species of the genus *Bacillus*.

In addition, it is important to remember the correct storage conditions for raw milk before further processing. Awasti et al. [53] conducted a study on strains of *Bacillus licheniformis*, which was also present in our samples. Their results indicate that factors such as temperature and storage time of raw milk affected changes in the growth of *Bacillus* sp. Changes occurred in the activity of spore production and spore germination, as well as in the proliferation of bacterial cells. According to the authors, storing raw milk for no more than 72 h at 8 °C can ensure that bacterial populations do not increase by 1.0 log CFU/mL. Increased temperature and extended storage can result in the development of potentially pathogenic microorganisms, including *Bacillus* sp.

Our research has some limitations which need to be addressed here. VITEK^®^MS (bioMérieux, Marcy l’Etoile, France) can not distinguish some species because of high similarities among them, including *Bacillus fordii* and *Bacillus fortis*, identified as *B. fordii*/*B. fortis*; *Bacillus subtilis*, *Bacillus amyloliquefaciens*, and *Bacillus vallismortis*, identified as *B. subtilis*/*amyloliquefaciens*/*vallismortis*; and members of the *B. cereus* group, which are identified as a group. The *Bacillus cereus* group includes several species, phylogenetically organized into three broad clades. *Bacillus cereus sensu stricto* and *Bacillus thuringiensis* occur in all clades. In the first clade are *B. anthracis* and *B. wiedmannii*. The second clade includes *B. mycoides*, *B. pseudomycoides*, *B. toyonensis*, *B. cytotoxicus*, and *B. weihenstephanensis* (now classified as *B. mycoides*) [54,55]. The third clade includes *B. bingmayongensis*, *B. gaemokensis*, and *B. manliponensis*. In the past, a number of studies have been carried out regarding the possibility of using MALDI-TOF MS for the species identification of members of the *B. cereus* group. Undoubtedly, MALDI-TOF MS has a large diagnostic potential, however, its limitation is the fact that, at the species level, the obtained mass spectra are almost identical and distinguishing them is more complicated, which makes it difficult to identify species within closely related microorganisms within the group of *B. cereus* [54].

## 5. Conclusions

The conducted research showed the presence of representatives of *Bacillus* spp. in raw milk. We found that all isolates were sensitive to chloramphenicol and meropenem. In addition, the *B. cereus* group strains were mostly sensitive to multiple antibiotics such as vancomycin, gentamicin, amikacin, and trimethoprim/sulfamethoxazole. However, most of them were identified as multidrug resistant with a high percentage of resistance to ampicillin, cefotaxime, rifampicin, and norfloxacin. The high level of multidrug resistance observed in *B. licheniformis* strains should also be considered at high risk. In contrast, the *B. subtilis* group strains showed a high percentage of resistance to norfloxacin but a low value of the multiple antibiotic resistance (MAR) index. The obtained results confirm the need for further research on *Bacillus* spp. present in raw milk in order to prevent the spread of antibiotic resistance among human pathogenic strains, which is a growing public health problem.

## Figures and Tables

**Table 1:** Antimicrobial susceptibility profile of *Bacillus* spp.

	No. (%) of Isolates	Antibiotic Profiles	MAR * Index
*B. cereus* group (*n* = 35)	1 (2.9%)	NOR	0.08
	1 (2.9%)	RD, NOR	0.17
	2 (5.7%)	AMP, CTX	0.17
	1 (2.9%)	AMP, CTX, NOR	0.25
	8 (22.9%)	AMP, CTX, RD	0.25
	1 (2.9%)	DA, CTX, NOR	0.25
	10 (28.6%)	AMP, CTX, RD, NOR	0.33
	2 (5.7%)	AMP, CTX, RD, SXT	0.33
	1 (2.9%)	AMP, DA, CTX, NOR	0.33
	1 (2.9%)	AMP, CTX, RD, SXT, NOR	0.42
	1 (2.9%)	CN, DA, E, CTX, NOR	0.42
	2 (5.7%)	AMP, DA, CTX, RD, NOR	0.42
	1 (2.9%)	AMP, DA, CTX, RD, SXT, NOR	0.5
	1 (2.9%)	CN, AMP, DA, CTX, RD, NOR	0.5
	1 (2.9%)	CN, AMP, E, CTX, RD, NOR	0.5
	1 (2.9%)	AK, AMP, DA, E, VA, CTX, RD, NOR	0.67
*B. licheniformis* (*n* = 7)	2 (28.6%)	DA, NOR	0.17
	1 (14.3%)	DA, CTX, NOR	0.25
	1 (14.3%)	DA, E, CTX	0.25
	1 (14.3%)	AK, DA, E, CTX	0.33
	1 (14.3%)	CN, AMP, CTX, RD, SXT, NOR	0.5
	1 (14.3%)	-	-
*B. pumilus* (*n* = 16)	5 (31.3%)	CTX	0.08
	4 (25.0%)	CTX, NOR	0.17
	2 (12.5%)	DA, CTX	0.17
	1 (6.3%)	AMP, CTX, RD	0.25
	1 (6.3%)	DA, CTX, NOR	0.25
	2 (12.5%)	DA, CTX, RD, NOR	0.33
	1 (6.3%)	-	-
*B. subtilis* group (*n* = 29)	12 (41.4%)	NOR	0.08
	1 (3.4%)	RD	0.08
	6 (20.7%)	RD, NOR	0.17
	1 (3,4%)	CN, NOR	0.17
	1 (3.4%)	AMP, CTX, RD	0.25
	8 (27.6%)	-	-
*Bacillus* sp. (*n* = 3)	1 (33.3%)	NOR	0.08
	1 (33.3%)	AMP, CTX, RD, NOR	0.33
	1 (33.3%)	-	-

* MAR—Multiple antimicrobial resistance index. CN—gentamicin, AK—amikacin, AMP—ampicillin, DA—clindamycin, E—erythromycin, VA—vancomycin, CTX—cefotaxime, RD—rifampicin, SXT—trimethoprim/sulfamethoxazole, and NOR—norfloxacin.

## Data Availability

Not applicable.
